# A common partitivirus infection in United States and Czech Republic isolates of bat white-nose syndrome fungal pathogen *Pseudogymnoascus destructans*

**DOI:** 10.1038/s41598-020-70375-6

**Published:** 2020-08-17

**Authors:** Ping Ren, Sunanda S. Rajkumar, Tao Zhang, Haixin Sui, Paul S. Masters, Natalia Martinkova, Alena Kubátová, Jiri Pikula, Sudha Chaturvedi, Vishnu Chaturvedi

**Affiliations:** 1 Mycology Laboratory, Wadsworth Center, New York State Department of Health, Albany, NY USA; 2 Present Address: Department of Pathology, University of Texas Medical Branch, Galveston, TX USA; 3 Institute of Medicinal Biotechnology, Chinese Academy of Medical Sciences and Peking Union Medical College, Beijing, People’s Republic of China; 4 Cellular and Molecular Basis of Diseases Laboratory, Wadsworth Center, New York State Department of Health, Albany, NY USA; 5 Department of Biomedical Sciences, University of Albany School of Public Health, Albany, NY USA; 6 Viral Replication and Vector Biology Laboratory, Wadsworth Center, New York State Department of Health, Albany, NY USA; 7 Institute of Vertebrate Biology, Czech Academy of Sciences, Brno, Czech Republic; 8 Department of Botany, Faculty of Science, Charles University in Prague, Praha, Czech Republic; 9 Department of Ecology and Diseases of Zoo Animals, Game, Fish and Bees, University of Veterinary and Pharmaceutical Sciences, Brno, Czech Republic; 10 Present Address: ICMR Medical Research Institute, Puducherry, India

**Keywords:** Fungi, Viral epidemiology

## Abstract

The psychrophilic (cold-loving) fungus *Pseudogymnoascus destructans* was discovered more than a decade ago to be the pathogen responsible for white-nose syndrome, an emerging disease of North American bats causing unprecedented population declines. The same species of fungus is found in Europe but without associated mortality in bats. We found *P. destructans* was infected with a mycovirus [named Pseudogymnoascus destructans partitivirus 1 (PdPV-1)]. The virus is bipartite, containing two double-stranded RNA (dsRNA) segments designated as dsRNA1 and dsRNA2. The cDNA sequences revealed that dsRNA1 dsRNA is 1,683 bp in length with an open reading frame (ORF) that encodes 539 amino acids (molecular mass of 62.7 kDa); dsRNA2 dsRNA is 1,524 bp in length with an ORF that encodes 434 amino acids (molecular mass of 46.9 kDa). The dsRNA1 ORF contains motifs representative of RNA-dependent RNA polymerase (RdRp), whereas the dsRNA2 ORF sequence showed homology with the putative capsid proteins (CPs) of mycoviruses. Phylogenetic analyses with PdPV-1 RdRp and CP sequences indicated that both segments constitute the genome of a novel virus in the family *Partitiviridae*. The purified virions were isometric with an estimated diameter of 33 nm. Reverse transcription PCR (RT-PCR) and sequencing revealed that all US isolates and a subset of Czech Republic isolates of *P. destructans* were infected with PdPV-1. However, PdPV-1 appears to be not widely dispersed in the fungal genus *Pseudogymnoascus*, as non-pathogenic fungi *P. appendiculatus* (1 isolate) and *P. roseus* (6 isolates) tested negative. *P. destructans* PdPV-1 could be a valuable tool to investigate fungal biogeography and the host–pathogen interactions in bat WNS.

## Introduction

The fungus *Pseudogymnoascus destructans* (previously named as *Geomyces destructans*) is a psychrophilic (cold-loving) fungus responsible for the white-nose syndrome (WNS) in bat populations in North America^1–3^. This newly discovered pathogen has directly or indirectly caused the death of more than 5.7 million bats since 2006^4,5^. *P. destructans* was found to infect at least eleven species of bats, of which seven species exhibited disease symptoms upon infection. The infected bat species include the endangered Indiana bat (*Myotis sodalis*) and the grey bat (*Myotis grisescens*) https://www.whitenosesyndrome.org/about/bats-affected-wns^6^.

*P. destructans* grows as an opportunistic pathogen on bat skin during bat hibernation in caves. It also persists in the cave environment as a saprotroph^7–9^. The optimal temperature of growth for this fungus is around 15 °C. It produces brown and grey colonies, secretes a brownish pigment, and reproduces asexually via asymmetrically curved conidia when cultured on Sabouraud dextrose agar (SAB)^3^. Although *P. destructans* isolates analyzed to date from the US and Canada represent a clonal population, variations were reported among isolates^10–12^. Despite apparent genetic homogeneity, phenotypic variations were reported among North American *P. destructans* isolates, specifically in the mycelial growth rate, exudate production, pigment formation, and diffusion into agar media^12^. The underlying cause(s) for the phenotypic variations remain to be discovered. Viral infection could be a potential reason for the variation in fungal phenotypic expression^13,14^.

Viruses that infect fungi (mycoviruses) are rather common and mostly latent^15,16^. The majority of mycoviruses are dsRNA-containing isometric particles, although ssRNA and ssDNA mycoviruses have also been recognized. Three well-studied mycovirus systems include (i) yeast killer toxins in *Saccharomyces cerevisiae* and non-conventional yeasts^17^, (ii) hypovirulence in *Cryphonectria parasitica* and *Sclerotinia sclerotiorum*, the causal agents of Chestnut blight and white mold diseases on hundreds of plant species, respectively^18–21^, and (iii) a symbiotic role in conferring heat tolerance to fungal endophytes of grasses^22^. There are reports linking mycovirus infection to phenotypic changes in the fungal host^23^.

Recently, dsRNA mycoviruses in the human pathogens *Aspergillus fumigatus* and *Talaromyces marneffei* were found to cause hypervirulence^24,25^. Mycoviruses could also provide a ‘phylogenomics window’ as their evolution showed a strong co-divergence with their fungal hosts^26^. We hypothesized that the origin, evolution, and virulence of *P. destructans* could be investigated by focusing on mycoviruses. The current study summarizes the molecular characterization of a virus [named Pseudogymnoascus destructans partitivirus 1 (PdPV-1)] that infects *P. destructans*. All US and a few Czech Republic isolates tested positive for PdPV-1. PdPV-1 appears to be host-specific to *P. destructans,* as closely related non-pathogenic fungi *P. appendiculatus* and *P. roseus*^27^ tested negative for the mycovirus. The results indicated that PdPV-1 represents a common feature among all US and some Czech Republic isolates of *P. destructans* and could be used to further investigate fungal biogeography and the host–pathogen interactions in bat WNS.

## Materials and methods

### Fungal isolates

*P. destructans* isolate (MYC80251) has been described previously^3^. *P. destructans* isolates and other *Pseudogymnoascus* species listed in Table 1, were maintained on SAB agar.

**Table 1:** Fungal isolates used in this study.

Fungal isolate	Location	Source	Viral RNA detection	GenBank accession#	
				RdRp	Capsid
***Pseudogymnoascus destructans***					
MYC80251	Albany, New York	*Myotis lucifugus*	Yes	KP128044 MK789667*	KP128045 MK789674*
PESU14	Avery, North Carolina	*Perimyotis subflavus*	Yes	MN990689	MN990699
LBB17	Lawrence, Ohio	*Myotis lucifugus*	Yes	MN990690	MN990700
PESU8	Greenbrier, West Virginia	*Perimyotis subflavus*	Yes	MN990691	MN990701
LBB11	Woodward, Pennsylvania	*Myotis lucifugus*	Yes	MN990692	MN990702
M2335	Tompkins, New York	*Myotis lucifugus*	Yes	MN990693	MN990703
M2337	Erie, New York	*Myotis lucifugus*	Yes	MN990694	MN990704
M2339	Ulster, New York	*Myotis lucifugus*	Yes	MN990695	MN990705
HA-8-2	Hailes Cave, New York	Cave wall swab	Yes	MN990696	MN990706
VTG1-5-1	Greely Mine, Vermont	Soil	Yes	MN990697	MN990707
AC-3-3	Aelous Cave, Vermont	Soil	Yes	MN990698	MN990708
CCF3937	Malá Amerika mine, Czech Republic	*Myotis myotis*	No		
CCF3938	Solenice tunnel, Czech Republic	*Myotis myotis*	Yes	KY609331 MK789668*	KY609337 MK789675*
CCF3939	Solenice tunnel, Czech Republic	*Myotis myotis*	Yes	MK789669*	MK789676*
CCF3941	Malá Amerika mine, Czech Republic	*Myotis myotis*	Yes	KY609332 MK789670*	KY609338 MK789677*
CCF3944	Nový Knín, Czech Republic	*Myotis myotis*	No		
CCF4103	Herlíkovice tunnel, Czech Republic	*Myotis myotis*	No		
CCF4124	Albeřická/Bischofova cave, Czech Republic	*Plecotus auritus*	No		
CCF4125	Albeřická/Bischofova cave, Czech Republic	*Myotis myotis*	No		
CCF4126	Portál tunnel, Czech Republic	*Myotis myotis*	No		
CCF4127	Herlíkovice tunnel, Czech Republic	*Myotis myotis*	Yes	KY609329 MK789671*	KY609335 MK789678*
CCF4129	Pístov cellar, Czech Republic	*Myotis myotis*	Yes	MK789672*	MK789679*
CCF4130	Fučná-Otov tunnel, Czech Republic	*Myotis myotis*	Yes	KY609328 MK789673*	KY609334 MK789680*
M3695	Malá Morávka mine, Czech Republic	*Myotis myotis*	Yes	KY609330	KY609336
M3696	Kateřinská cave, Czech Republic	*Myotis bechsteinii*	No		
M3697	Malá Morávka mine, Czech Republic	*Myotis emarginatus*	No		
M3698	Kateřinská cave, Czech Republic	*Myotis nattereri*	Yes	KY609333	KY609339
M3699	Malá Morávka mine, Czech Republic	*Myotis daubentonii*	No		
M3701	Sloupsko-Šošůvské cave, Czech Republic	*Myotis myotis*	No		
***Pseudogymnoascus roseus***					
3-VT-5	Vermont	Hibernacular soil	No		
5-NY-6	New York	Hibernacular soil	No		
5-NY-8	New York	Hibernacular soil	No		
5-NY-9	New York	Hibernacular soil	No		
WSF-3629	Wisconsin	Amorphus peat	No		
UAMH1658	Canada	Sphagnum bog	No		
***Pseudogymnoascus appendiculatus***					
UAMH10510	Canada	Wood bait block, Sphagnum bog	No		

*GenBank accession numbers for sequences that were determined in the laboratory in Beijing, China; all other accession numbers are for sequences determined in the Albany, NY laboratory.

### dsRNA extraction

*P. destructans* was grown in a stationary culture in potato dextrose broth (PDB) at 15 °C for 1–2 months in 500-ml flasks. About 1 g wet weight mycelium was harvested and ground to powder under liquid nitrogen with a mortar and pestle. The powder was collected and suspended in 1 ml extraction buffer (150 mM sodium acetate, pH 5.0, 100 mM LiCl, 4% sodium dodecyl sulfate, 10 mM EDTA, pH 8.0, and 20 mM β-mercaptoethanol) and incubated on ice for 10 min. Total nucleic acids were obtained by standard phenol–chloroform extraction followed by precipitation with LiCl and isopropanol. ssRNA and DNA were removed by treatment with S1 nuclease (Life Technologies; Carlsbad, CA) in buffer containing 30 mM sodium acetate (pH 4.6), 50 mM NaCl, 1 mM zinc acetate, 0.5 mg/ml heat-denatured DNA, and 5% (v/v) glycerol at 37 °C for 10 min, followed by incubation with DNase I (Epicentre; Madison, WI) at 37 °C for 30 min. The reaction was extracted with an equal volume of Tris–EDTA–saturated phenol–chloroform–isoamyl alcohol (25:24:1), followed by extraction with an equal volume of chloroform–isoamyl alcohol (24:1). Undigested nucleic acid was precipitated with 2 volumes of absolute ethanol at − 20 °C overnight and recovered by centrifugation for 15 min at 10,000×*g*. Then the pellet was rinsed with 70% ethanol, air-dried and resuspended in 40 µl of RNase-free water. The extracted material was electrophoresed on a 1% agarose gel, stained with ethidium bromide and visualized by UV transillumination. To confirm the dsRNA nature of the remaining material, a 15-µg sample was treated with RNase III and RNase A (Life Technologies) at 37 °C for 1 h. Denaturing polyacrylamide gel electrophoresis of the purified dsRNA was used to determine the size of the two RNA bands.

### cDNA synthesis and sequence analysis

Purified dsRNA fractions (1–5 µg) containing 2 segments were denatured in 90% dimethyl sulfoxide (DMSO) at 65 °C for 20 min in the presence of random hexadeoxynucleotide and quickly chilled on ice. The RNA-primer mixture was precipitated with ethanol and resuspended in 11 µl RNase-free water. First-strand cDNA was synthesized using M-MLV reverse transcriptase (Life Technologies), based on the manufacturer’s instructions. Briefly, the denatured dsRNA with the random hexadeoxynucleotide were mixed with dNTPs, 5 × First-Strand Buffer, 0.1 M DTT, and RNaseOUT Recombinant Ribonuclease Inhibitor (40 units/µl) and incubated at 37 °C for 2 min. After adding 1 µl (200 units) of M-MLV RT, the mixture was incubated at 25 °C for 10 min, followed by 37 °C for 50 min. The reaction was inactivated by heating at 70 °C for 15 min. The resulting cDNA was amplified with random primers and the variously sized PCR products were cloned in TOPO TA cloning vector. A series of overlapping cDNA clones were obtained from the sequencing of the positive clones (S1 Table). Determination of the ends of each dsRNA was done using FirstChoice^®^ RLM-RACE Kit (Life Technologies). All the sequence contigs were assembled by Sequencher 4.8 (Gene Codes Co.; Ann Arbor, MI). Conserved sequences in GenBank were identified by searches using the tblastx program. Multiple sequence alignments for the two dsRNA segments were carried out using ClustalW analysis by MacVector 7.2 (Accelrys Inc.; Cary, NC). The maximum likelihood phylogenetic trees were generated by MEGA 6.0^28^ (S2 Table).

### Virus purification

Approximately 60 g wet weight fungal mycelium were collected and ground to powder as described above. The homogenate was mixed with extraction buffer (0.1 M sodium phosphate, pH 7.4, containing 0.1% β-mercaptoethanol) at a volume of 5 ml/g of wet mycelium. Following the addition of an equal volume of chloroform, the suspension was vortexed extensively, and the resulting emulsion was broken by centrifugation in a Sorvall GSA rotor at 10,000×*g* for 20 min. The upper aqueous layer was then mixed thoroughly with 0.5 volumes of 30% polyethylene glycol 8000 (PEG) in 0.85% NaCl and held on ice for 1 h. The PEG precipitate was pelleted in a Sorvall GSA rotor at 16,000×*g* at 4 °C for 30 min and resuspended in 0.1 M sodium phosphate, pH 7.4. After centrifugation at 23,000×*g* at 4 °C for 20 min to remove unsuspended debris, virus was collected by ultracentrifugation of the supernatant in a Beckman SW41 rotor at 76,000×*g*, 4 °C for 2 h. The pelleted virus was resuspended in a total of 4 ml 0.1 M sodium phosphate buffer and purified by loading the viral suspension onto pre-formed gradients of 10–50% (w/v) sucrose in 0.1 M sodium phosphate buffer and centrifuging at 76,000×*g*, 4 °C overnight. The collected virus fractions were diluted in 0.1 M sodium phosphate buffer and preserved at 4 °C for immediate electron microscopy^29–31^.

### Negatively staining electron microscopy

Two µl of the virus solution was placed on a glow-discharged copper grid covered with a continuous carbon film. After 1 min of adsorption, the grid was washed with pure water for several seconds and stained with 3 µl of 2% (w/v) uranyl acetate solution for 1 min. The staining solution was blotted away with Whatman No. 1 filter paper. The grid was air-dried completely before it was examined in a JEOL JEM-1400 electron microscope operating at 120 keV. The micrographs were recorded at various magnifications using a 4K × 4K CMOS camera (TVIPS F-416).

### Reverse transcription PCR (RT-PCR) assay

First-strand cDNA synthesis was performed as described earlier and followed by PCR using primer pairs V2085/V2090 (40 cycles of 94 °C for 30 s, 57.5 °C for 30 s, and 72 °C for 1 min) and V2168/V2164 (40 cycles of 94 °C for 30 s, 56.5 °C for 30 s, and 72 °C for 1 min) to amplify partial dsRNA1 and dsRNA2 segments with expected fragment lengths of 828 bp and 613 bp, respectively (S1 Table). PCR products were sequenced using the same primers and aligned to the prototype PdPV-1 sequence from MYC80251 to verify the identities of the amplicons obtained.

### Independent laboratory confirmatory analysis

In order to further confirm our findings, one of us (TZ) imported four of the original six positive Czech *P. destructans* isolates, as well as two isolates not previously tested in the US laboratory, directly from the Czech Republic to China. Additionally, the MYC80251 isolate was imported from the US to China. RNA extraction, RT-PCR, and sequencing analysis of these isolates were carried out with primers, probes, and fresh reagents in a laboratory in Beijing, China that had no prior history of *P. destructans* investigations.

## Results

### Isolation and characterization of dsRNA from *P. destructans*

**Figure 1:**
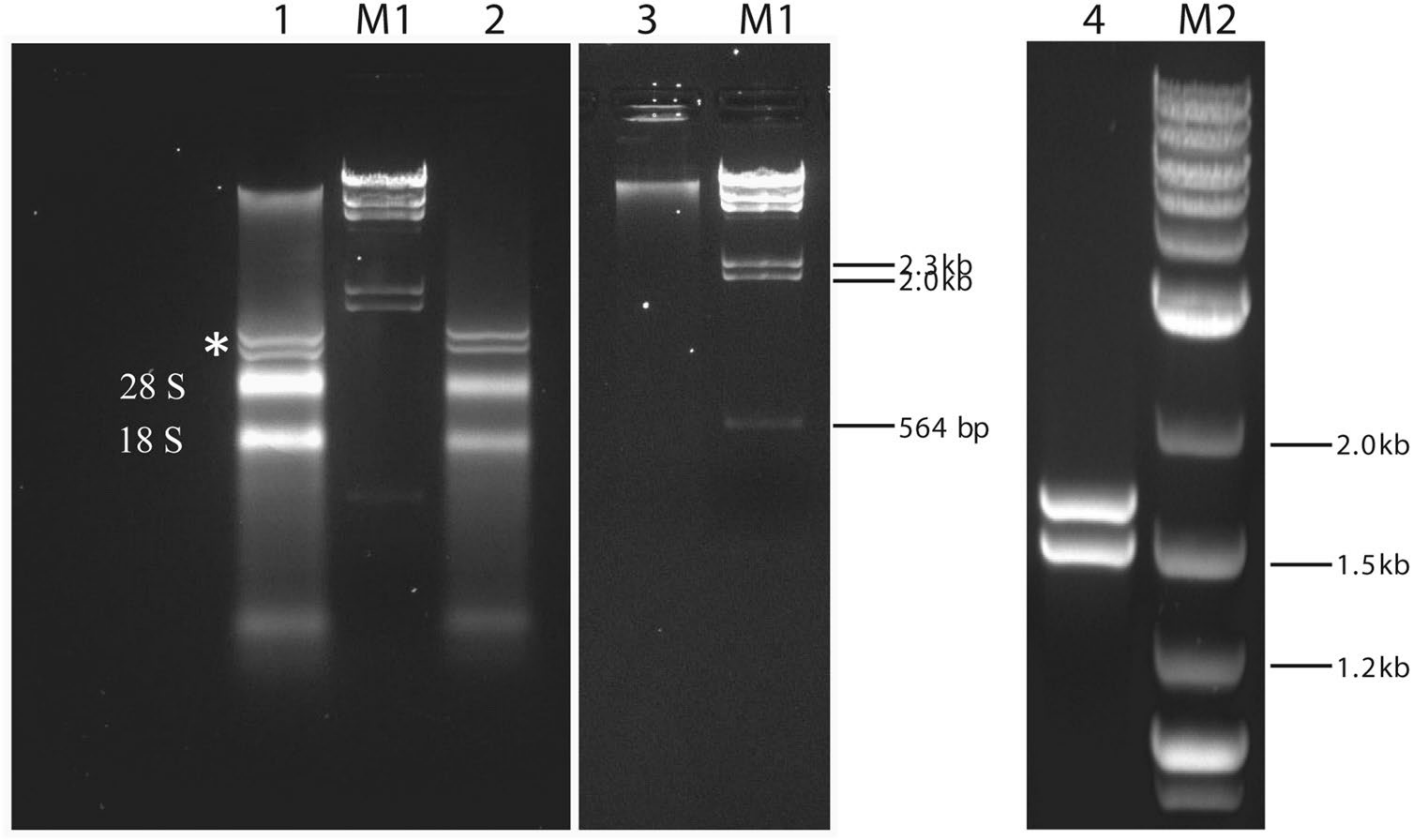
Analysis of nucleic acids extracted from *P. destructans* by differential endonuclease sensitivity or resistance. Samples were digested with the indicated enzymes, and undigested species were separated by electrophoresis in 1% native agarose gels. Lane 1: total *P. destructans* nucleic acids; the 28 S and 18 S ribosomal RNAs are indicated, and the two unknown, more slowly migrating bands are labeled with an asterisk. Lane 2: total nucleic acids following digestion with DNase I. Lane 3: total nucleic acids following digestion with both RNase III and RNase A. Lane 4: total nucleic acids following digestion with both S1 nuclease and DNase I. Size markers: lanes M1, lambda DNA-HindIII digest; lane M2, 2-log DNA ladder (New England BioLabs).

**Figure 2:**
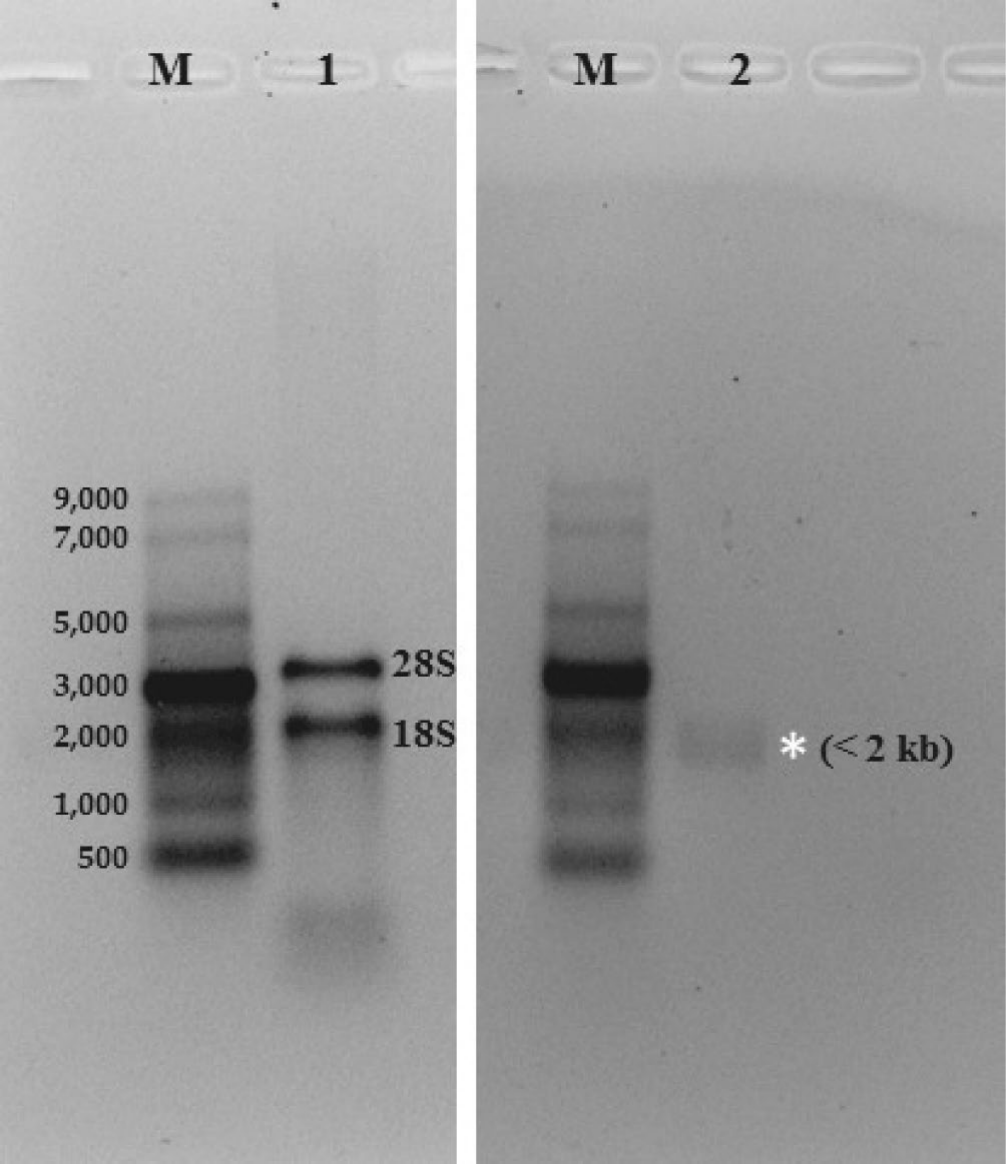
Estimation of the size of the dsRNA species in nucleic acids extracted from *P. destructans*. Samples were analyzed by electrophoresis in denaturing polyacrylamide gels. Lane 1: total RNA; the 28 S and 18 S ribosomal RNAs are indicated. Lane 2: total RNA digested with RNase A prior to reisolation and denaturation. Size marker (lanes M): ssRNA ladder (in nucleotides).

Total nucleic acids extracted from *P. destructans* isolate MYC80251 migrated as multiple bands when analyzed by native agarose gel electrophoresis (Fig. 1, lane 1). The most slowly migrating set of bands was found to be removed by treatment with DNase I, showing that these corresponded to fungal genomic DNA (Fig. 1, lane 2). Conversely, only the high molecular weight fungal genomic DNA bands remained following treatment of total *P. destructans* nucleic acids with a combination of RNase III, a dsRNA-specific endoribonuclease, and RNase A, a pancreatic ribonuclease that cleaves ssRNA (Fig. 1, lane 3). This demonstrated that all of the other bands were RNA species. The two dominant RNA bands were most likely fungal 28S and 18S ribosomal RNAs. Migrating more slowly than the rRNAs were two prominent discrete bands of unknown identities (labeled by an asterisk in lane 1). To determine the nature of these bands, total extracted nucleic acids were digested with a combination of S1 nuclease (a single-strand-specific endonuclease) and DNase I. This treatment abolished all RNA and DNA species except the unknown doublet of bands (Fig. 1, lane 4). Collectively, these results strongly indicated that the unknown bands were double-stranded RNA (dsRNA), most likely derived from a fungal virus. Because the analysis in Fig. 1 was carried out in native agarose gels, the mobilities of RNA species were compared to those of dsDNA size markers. To more accurately gauge the sizes of the two dsRNA bands, they were analyzed by denaturing polyacrylamide gel electrophoresis in comparison to ssRNA size markers. In this type of gel, the denatured dsRNAs were almost totally masked by the presence of the 18S rRNA band in a sample of total *P. destructans* RNA (Fig. 2, lane 1). However, removal of rRNAs by RNase A treatment of total RNA prior to reisolation, denaturation and electrophoresis allowed us to estimate that the dsRNA species fell in the size range of 1.5–2.0 kb (Fig. 2, lane 2, labeled by asterisk).

### Electron microscopy (EM)

**Figure 3:**
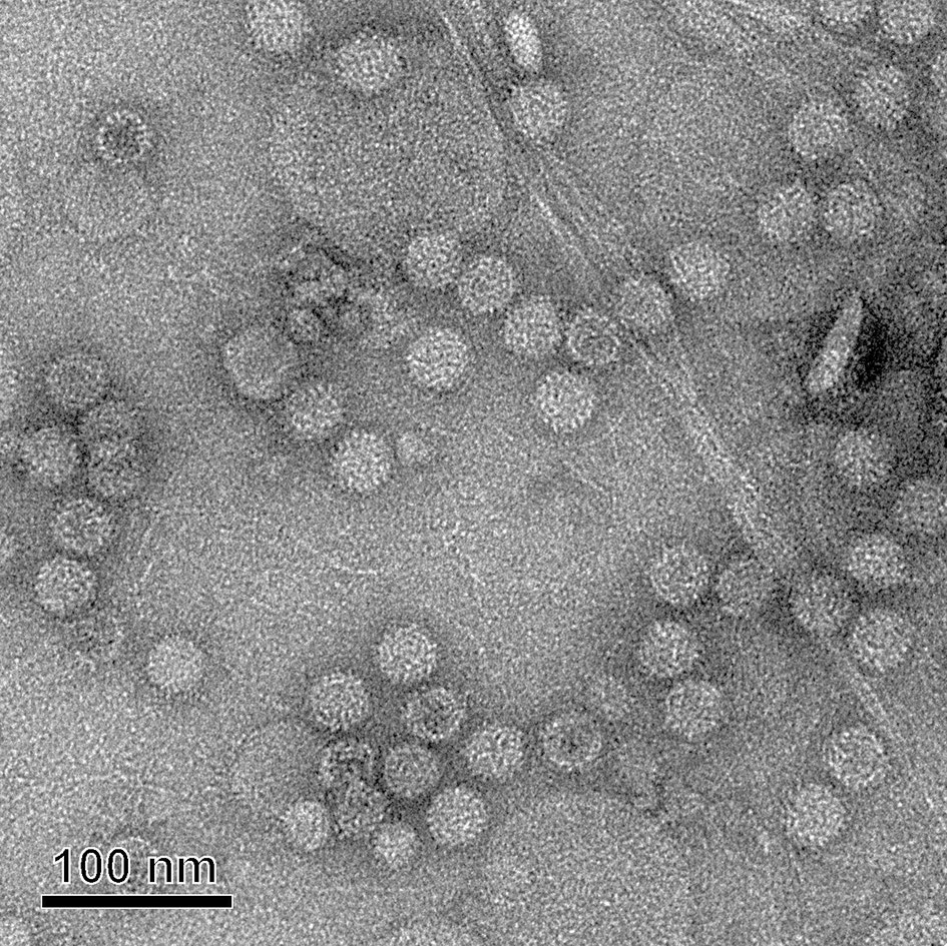
Electron microscopy of purified PdPV-1. Isometric viral particles with an estimated diameter of 33 nm were observed. The bar represents 100 nm.

The presence of viral particles in *P. destructans* was confirmed by transmission EM. Viral particles (named as PdPV-1) were purified from mycelia of *P. destructans* MYC80251 by homogenization, PEG precipitation, and sucrose density gradient centrifugation. Large amounts of isometric viral particles were observed under EM. The diameter of the virions was estimated to be about 33 nm (Fig. 3).

### Sequence and phylogenetic analysis

**Figure 4:**
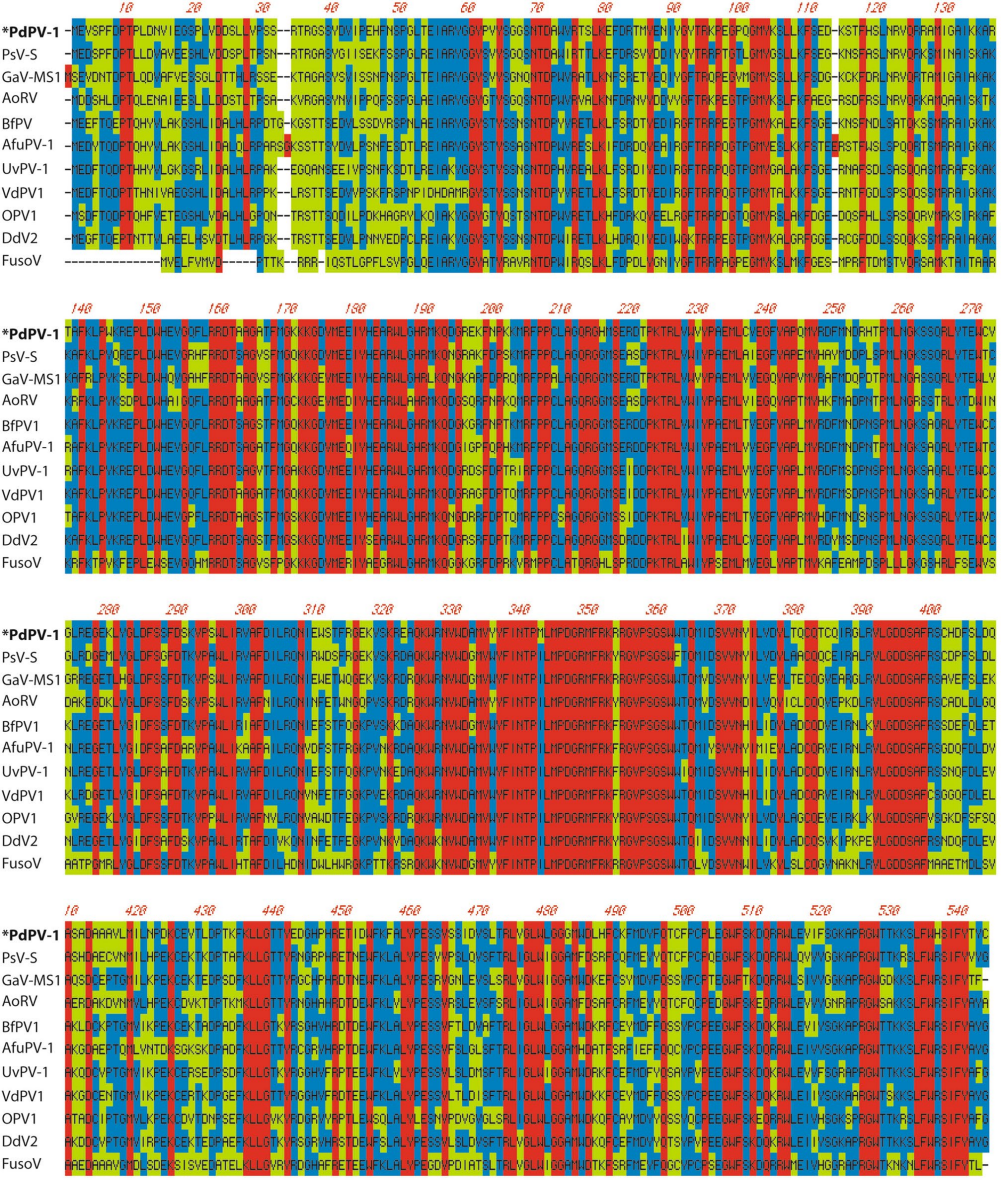
Comparison of the amino acid sequences of putative RdRp of the Pseudogymnoascus destructans virus (PdPV-1), Penicillium stoloniferum virus S (PsV-S), Gremmeniella abietina virus MS1 (GaV-MS1), Aspergillus ochraceus virus (AoV), Botryotinia fuckeliana partitivirus-1 (BfPV1), Aspergillus fumigatus partitivirus-1 (AfuPV-1), Ustilaginoidea virens partitivirus 1 (UvPV-1), Verticillium dahliae partitivirus 1 (VdPV1), Ophiostoma partitivirus (OPV1), Discula destructiva virus 2 (DdV2), and Fusarium solani virus 1 (FusoV). Red: 100% identity; Blue: consensus match; Green: mismatch.

**Figure 5:**
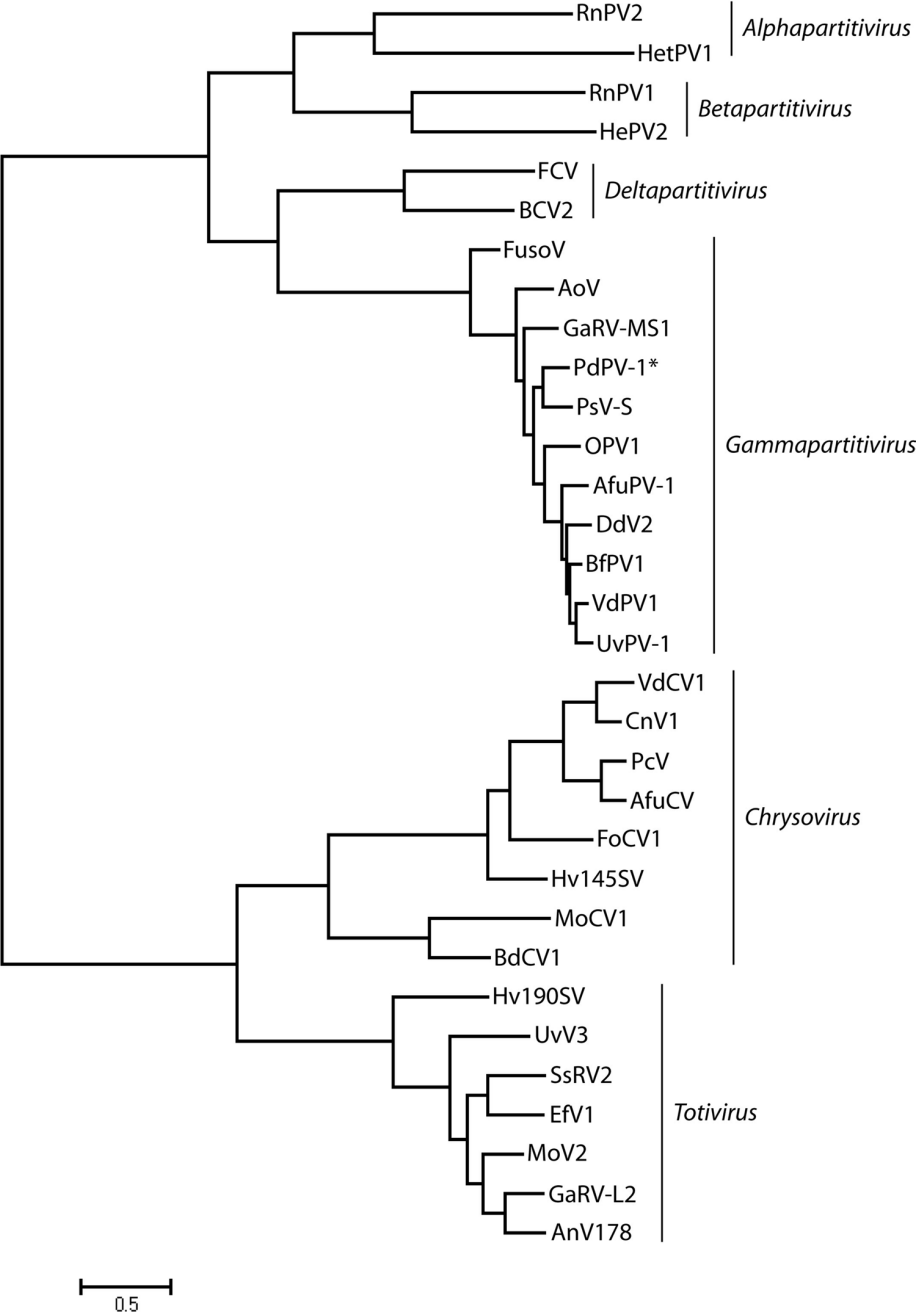
Phylogenetic analysis of PdPV-1. Maximum likelihood phylogenetic tree based on RdRp amino acid sequences of representative members of the families *Partitiviridae *(including genera *Alphapartitivirus, Betapartitivirus, Deltapartitivirus,* and *Gammapartitivirus*),* Totiviridae,* and *Chrysoviridae* were constructed using the program MEGA 6 (GenBank accession numbers are in the Table S2).

The complete sequences of the two dsRNA genome segments of PdPV-1 were obtained by sequencing of a library of cDNA clones, gap-filling reverse transcription PCR, and RLM-RACE. The large segment dsRNA1 comprised 1,683 bp with 46% GC content. The dsRNA1 segment contains an open reading frame (ORF) that encodes 539 amino acids with a molecular mass of approximately 62.7 kDa (GenBank Accession number: KP128044). The small segment dsRNA2 comprised 1,524 bp with 49% GC content. The dsRNA2 segment contains an ORF that encodes 434 amino acids with a molecular mass of approximately 46.9 kDa (GenBank Accession number: KP128045). Both ORFs were identified on the positive strand of each dsRNA segment. The negative strands did not contain any significant ORFs that are longer than 82 amino acids. The search for similar deduced amino-acid sequences in GenBank revealed that the ORFs of dsRNA dsRNA1 and dsRNA2 have significant similarities to the putative RNA-dependent RNA polymerase (RdRp) and the capsid protein (CP), respectively, of viruses from the family *Partitiviridae* genus *Gammapartitivirus*^32^*.* These include Penicillium stoloniferum virus S (PsV-S), Aspergillus fumigatus partitivirus-1 (AfuPV-1), Aspergillus ochraceus virus (AoV), Botryotinia fuckeliana partitivirus 1 (BfPV1), Discula destructiva virus 2 (DdV2), Fusarium solani virus 1 (FusoV), Gremmeniella abietina virus MS1 (GaV-MS1), Ophiostoma partitivirus (OPV1), Ustilaginoidea virens partitivirus (UvPV-1), and Verticillium dahliae partitivirus 1 (VdPV1) (Fig. 4 and Fig. S1). Specifically, the RdRp of PdPV-1 showed 76% identity with PsV-S RdRp, whereas the CP of PdPV-1 showed 67% identity with that of PsV-S. Homologies (amino acid identity in red and consensus match in blue) were much higher within the core motif regions of the RdRp (amino acids 174–491) of PdPV-1, than elsewhere in the molecule (Fig. 4). Phylogenetic analyses were performed by using the RdRp and CP sequences of the various mycoviruses such as alphapartitiviruses, betapartitivirues, deltapartitiviruses, gammapartitiviruses, totiviruses, and chrysoviruses. Phylogenetic trees derived from both RdRp and CP sequences exhibited three major branches and suggested that PdPV-1 is a member of the genus *Gammapartitivirus* in the family *Partitiviridae* (Fig. 5 and Fig. S2). In addition, phylogenetic analyses of the putative RdRp and CP of PdPV-1 showed that PdPV-1 was most closely related to the gammapartitivirus PsV-S.

We found that the 5′ untranslated regions (UTRs) of the plus strands of segments dsRNA1 and dsRNA2 were 8 and 60 nucleotides, respectively; whereas the 3′ UTRs of the plus strands of segments dsRNA1 and dsRNA2 were 54 and 159 nucleotides, respectively. In contrast, a separate study of PdPV-1^33^ obtained longer RLM-RACE reads than ours and was able to identify conserved 5′-terminal nucleotide sequence motifs in both genome segments, a feature that is common to the majority of partitiviruses^29,34–36^. Additionally, as a consequence of the observed longer 5′ end of the dsRNA2 sequence, the deduced CP amino-acid sequence in that study^33^ had a 35-residue amino-terminal extension relative to the CP ORF that we determined. If confirmed, such an extension would make the PdPV-1 CP unique among the partitiviruses (Fig. S1). This apparent discrepancy remains to be resolved in the future by the direct characterization of the CP. Very recently, a PdPV-1 sequence was also assembled in a metagenomic analysis of fungal transcriptomic datasets^37^. Notably, other than the amino-terminal CP extension^33^, the PdPV-1 sequences obtained in all three studies were 100% identical in the dsRNA1 and dsRNA2 coding regions.

### Reverse transcription PCR (RT-PCR) screening of *P. destructans* isolates for the presence of PdPV-1

RT-PCR was performed to examine whether PdPV-1 exists in various *P. destructans* isolates from diverse sources. *P. destructans* isolates listed in Table 1 include those collected from fungus-infected bats in the caves from various geographic locations in the US and the Czech Republic, as well as those that were purified from environmental samples collected from caves or mines where bats with WNS hibernated. Closely related strains of *Pseudogymnoascus roseus* that were isolated from soil samples collected in bat hibernacula were also tested. *P. roseus* WSF-3629 was collected from Amorphus peat in Wisconsin^27^. *P. roseus* UAMH1658 and *P. appendiculatus* UAMH10510 were obtained from the University of Alberta Microfungus Collection and Herbarium. All these bat tissue samples and environmental isolates were subjected to total RNA extraction, followed by virus-specific RT-PCR detection and sequencing of RT-PCR product fragments. Both dsRNA1 and dsRNA2 segments of PdPV-1 were detected in RNA extracts of all *P. destructans* isolates from the US, irrespective of where they were collected, and their partial sequences (GenBank accession numbers: MN990689–MN990708) were 100% identical to those of our original PdPV-1 strain from MYC80251. Six out of sixteen *P. destructans* isolates collected in the Czech Republic were also found to be PdPV-1 positive. dsRNA1 and dsRNA2 segments of these six PdPV-1 positive isolates were partially sequenced (GenBank accession numbers: KY609328–KY609339), and we found that some of these PdPV-1 strains had polymorphisms in both their dsRNA1 and dsRNA2 segments, while others were identical to the sequences of PdPV-1 from the US *P. destructans* isolates. Additionally, examination of the closely related fungi *P. roseus* and *P. appendiculatus* showed no detectable dsRNA1 or dsRNA2 segment of PdPV-1 dsRNA (Table 1).

### Independent analysis of *P. destructans* isolates from the Czech Republic

To obtain independent verification of some of these findings, four of the Czech Republic isolates (CCF3938, CCF3941, CCF4127, and CCF4130) that had been found to be positive for PdPV-1 by testing in the US were imported directly from the Czech Republic to China and were tested in a laboratory in Beijing that had no prior history of work with *P. destructans*. These isolates, plus two previously untested Czech isolates (CCF3939 and CCF4129), were confirmed by RT-PCR and sequencing to harbor PdPV-1 (GenBank accession numbers: MK789668–MK789673 and MK789675–MK789680) (Table 1).The laboratory in Beijing also confirmed the presence of PdPV-1 in the US *P. destructans* isolate MYC80251 (GenBank accession numbers: MK789667 and MK789674) (Table 1).

## Discussion

Bat WNS continues to devastate the bat populations in the United States and Canada and therefore, there remains an urgent need to understand the origin and spread of this disease. In this study, we characterized mycovirus PdPV-1 infecting the WNS etiological fungus *P. destructans*. The identity of PdPV-1 was confirmed by (i) demonstration of its dsRNA content by differential sensitivity or resistance to DNA and RNA endonucleases; (ii) complete viral genome sequencing; (iii) nucleotide similarities of the dsRNA viral genome segments and phylogenetic alignment of their encoded proteins with mycoviruses from the family *Partitiviridae* genus *Gammapartitivirus*; (iv) TEM confirmation of isometric viral particles, and (v) host specificity of PdPV-1 for *P. destructans* and no detection of this virus in closely related *Pseudogymnoascus* species. Overall, PdPV-1 exhibited size, morphology, and nucleotide sequences typical of the fungal viruses in the family *Partitiviridae* genus *Gammapartitivirus*^32,38^.

These findings in our study (https://www.biorxiv.org/content/10.1101/059709v1) were obtained independently from and prior to a report by Thapa et al*.*^33^. In contrast to results contained in that publication, we found that some of the Czech Republic *P. destructans* isolates were infected with PdPV-1. To eliminate the possibility that these results were false-positives, virus testing was repeated separately using four of the original six positive isolates that were shipped directly to China from the Czech Republic. Testing in the laboratory in Beijing verified that these four isolates contained PdPV-1, as did two other isolates not previously tested in the US (Table 1). This confirmation of our results strongly supports our conclusion that PdPV-1 infection is not unique to the North American isolates of *P. destructans*. We do not know the basis for the discrepancy between the results of our laboratories and those of the other report^33^; notably, dsRNA1 (GenBank Accession number KP128044 and KY207543) and dsRNA2 (GenBank Accession number KP128045 and KY207544) from the two laboratories have 100% nucleotide identity, respectively. It is possible that it may be attributable to differences in the methods used for dsRNA extraction. Also, we note that the Czech *P. destructans* isolates that we tested were obtained from laboratories in the Czech Republic, whereas those analyzed by Thapa et al*.*^33^ were acquired from the Center for Forest Mycology Research in Madison, Wisconsin.

Both *P. destructans* and its closely related species of *P. roseus* have been isolated from the soil of WNS affected caves and mines^7,39^. PdPV-1 was only detected from the randomly selected samples of *P. destructans*, but not from *P. roseus*. The result showed that all US *P. destructans* isolates were infected with PdPV-1, no matter whether the samples were isolated from the bats with WNS or its environment in the US. Virus-free fungal isolates of *P. roseus* were not found on the bats with WNS although this fungal species was found in the same environmental reservoir as the WNS pathogen. The absence of PdPV-1 infection in other *Pseudogymnoascus* species, collected from the same bat hibernacula in the US, suggested a close viral association with *P. destructans*. The observed host-specificity of PdPV-1 was consistent with the narrow host ranges of mycoviruses due to severe bottlenecks in their horizontal transmission^16^. The above results raise two questions: (i) If PdPV-1 can infect *P. roseus*, will *P. roseus* with virus cause WNS? (ii) Can virus-free isolates of *P. destructans* infect bats to cause WNS? Additional studies are needed to address these questions.

The discovery of a dsRNA mycovirus in *P. destructans* is consistent with the wide occurrence of mycoviruses in fungi^15,40^. Mycovirus infection of fungi is usually asymptomatic^41^. However, mycovirus infection could alter the ability of plant-pathogenic fungi to cause diseases^23,42^. In plant-pathogenic fungi, mycovirus infections generally reduce fungal yield, attenuate mycelial growth, abolish female fertility in sexual crosses, decrease asexual sporulation, alter colony morphologies, modulate pigment production, and diminish the accumulation of specific metabolites. These effects lead to hypovirulence, an attenuation of the pathogenic outcome of fungal infection on the plant host^20^. The harmful effects of mycoviruses on fungal growth were exploited as beneficial functions in phytopathogen control^15,43,44^. On the contrary, virus infections of animal pathogenic fungi and protozoan pathogens showed hypervirulence^45^. A recent publication suggested that partitivirus infections of the dimorphic human fungal pathogen *Talaromyces marneffei* caused aberrant gene expression and hypervirulence in an animal model^25^. More in-depth experiments will be needed to discern the outcome of PdPV-1 infection on *P. destructans* phenotype and virulence. These investigations will be facilitated by the recent availability of a molecular tool kit and experimental infection model of *P. destructans*^46–51^.

In conclusion, mycoviruses are ubiquitous in fungi, while the connection between fungal phenotype and mycovirus presence is not always straightforward. In this study, we discovered the existence of the partitivirus PdPV-1 in *P. destructans* isolated from various species of bats with WNS or from their living environment in both the US and the Czech Republic. *P. destructans* PdPV-1 could be a valuable tool to investigate fungal biogeography and the host–pathogen interactions in bat WNS.

## Supplementary information

Supplementary Information.

## Data Availability

All nucleotide sequences generated in this study are deposited in the NCBI database.
