# Supplementary material for: A common partitivirus infection in United States and Czech Republic isolates of bat white-nose syndrome fungal pathogen Pseudogymnoascus destructans
Source: Sci Rep. 2020 Aug 17;10:13893. doi: 10.1038/s41598-020-70375-6 (PMC7431587; doi:10.1038/s41598-020-70375-6)
Supplement: Supplementary file 1 — Supplementary Information. [file 41598_2020_70375_MOESM1_ESM.docx]

**Supplementary information**

**A common partitivirus infection in United States and Czech Republic isolates of bat White-nose syndrome fungal pathogen *Pseudogymnoascus destructans***

Ping Ren, Sunanda S. Rajkumar, Tao Zhang, Haixin Sui, Paul S. Masters, Natalia Martinkova, Alena Kubátová, Jiri Pikula, Sudha Chaturvedi, Vishnu Chaturvedi*

**S1 Table. Oligonucleotide primers used in this study.**

| **Primer name** | **Sequences 5’ to 3’** | **Use in** |
| --- | --- | --- |
| M13 F | GTAAAACGACGGCCAGT | sequencing |
| M13 Rev | AACAGCTATGACCATG | sequencing |
| V2061 Rev | GAACTGACCTACTTCATGCCAG | sequencing |
| V2062 Rev | GAAGGTTGACTTGTCCTCTGAG | sequencing |
| V2063 F | CGATTCAGCGTTTCGTTCTTGC | RLM-RACE, sequencing |
| V2064 F | CTGGTTCAAGTTCGCGCTGTAC | RLM-RACE, sequencing |
| V2065 F | AGCCACAGTCAGATGGTACAGG | sequencing |
| V2066 F | TGTAACTCTCCGTGGTACGGAG | RLM-RACE, sequencing |
| V2067 Rev | ATGGGAATGTACTATCTGCTGG | RLM-RACE, sequencing |
| V2068 Rev | TCAGCATACTTCTGGTTACGAG | RLM-RACE, sequencing |
| V2085 F | AAAAGAATTCGACCGAACAATGTAC | RT-PCR, sequencing |
| V2086 Rev | ATTTATACATACCCTGCGGACCTT | RLM-RACE, sequencing |
| V2088 Rev | ACGGCGAAGGAACTGACCTA | RLM-RACE |
| V2089 F | GTTTGGGATGCTATGGTCTATTACTTT | sequencing |
| V2090 Rev | CCTCTCCTCTTACGGAACATTCTT | RT-PCR, sequencing |
| V2092 Rev | ATCGGGATTCAGTATCATCA | sequencing |
| V2094 Rev | AGACCCGCCGGAATACGA | RLM-RACE |
| V2096 Rev | GTTCCGATTGTTGGAGAGGA | Cloning, sequencing |
| V2097 Rev | CTCTTGATGCTCTCCCAAGC | sequencing |
| V2163 F | CCGAGGCATTGCTTCGTTTC | RT-PCR, sequencing |
| V2164 Rev | ACCCAGTCAAGGTCGAGTTC | RT-PCR, sequencing |
| V2165 Rev | CTTTTCACGACCGTCTTGCT | sequencing |
| V2168 F | TGACCAATGGGCAGTTTGGA | Cloning, RT-PCR, sequencing |
| V2186 F | TGCATGGTCAGAAGGTATTG | sequencing |
| V2210 Rev | TTGGAGCCACCTGAGTAAAC | sequencing |

**S2 Table. Accession number and other details of amino acid sequences used for phylogenetic analyses.**

| Mycovirus | Abbreviation | Amino acid sequences GenBank accession number | |
| --- | --- | --- | --- |
|  |  | for RdRp | for Capsid |
| *Aspergillus fumigatus* chrysovirus | AfuCV | CAX48749 | CAX48751 |
| *Aspergillus fumigatus* partitivirus-1 | AfuPV-1 | CAY25801 | CAZ61323 |
| *Aspergillus* mycovirus 178 | AnV178 | ABX79995 | ABX79994 |
| *Aspergillus ochraceous* virus | AoV | ABV30675 | ABV30676 |
| *Beet cryptic* virus 2 | BCV2 | ADP24757 | Not available |
| *Botryosphaeria dothidea* chrysovirus 1 | BdCV1 | AGZ84312 | AGZ84313 |
| *Botryotinia fuckeliana* partitivirus 1 | BfPV1 | CAM33266 | CAM33267 |
| *Cryphonectria nitschkei* chrysovirus 1 | CnV1 | ACT79256 | ACT79252 |
| *Discula destructive* virus 2 | DdV2 | AAK59379 | AAK59380 |
| *Epichloe festucae* virus 1 | EfV1 | CAK02788 | CAK02787 |
| *Fig cryptic* virus | FCV | CBW77436 | CBW77437 |
| *Fusarium oxysporum* chrysovirus | FoCV1 | ABQ53134 | ABQ58816 |
| *Fusarium solani* mycovirus | FusoV | BAA09520 | BAA09521 |
| *Gremmeniella abietina* RNA virus L2 | GaRV-L2 | YP_044807 | YP_044806 |
| *Gremmeniella abietina* RNA virus MS1 | GaRV-MS1 | AII16004 | AII16002 |
| *Helminthosporium victoriae* 145S virus | Hv145SV | YP_052858 | YP_052859 |
| *Helminthosporium victoriae* virus 190S | Hv190SV | NP_619670 | NP_619669 |
| *Heterobasidion* partitivirus 1 | HetPV1 | ADV15441 | ADV15442 |
| *Heterobasidion* partitivirus 2 | HetPV2 | ADL66906 | ADL66905 |
| *Magnaporthe oryzae* chrysovirus 1 | MoCV1 | YP_003858286 | Not available |
| *Magnaporthe oryzae* virus 2 | MoV2 | YP_001649206 | YP_001649205 |
| *Ophiostoma* partitivirus 1 | OPV1 | CAJ31886 | CAJ31887 |
| *Penicillium chrysogenum* virus | PcV | YP_392482 | YP_392481 |
| *Penicillicum stoloniferum* virus S | PsV-S | AAN86834 | AAN86835 |
| *Pseudogymnoascus destructans* virus 1 | PdV-1 | KP128044 | KP128045 |
| *Rosellinia necatrix* partitivirus 2 | RnPV1 | BAD98237 | BAD98238 |
| *Rosellinia necatrix* partitivirus 2 | RnPV2 | BAM78602 | BAK53192 |
| *Sphaeropsis sapinea* RNA virus 2 | SsRV2 | NP_047560 | NP_047559 |
| *Ustilaginoidea virens* partitivirus | UvPV-1 | AGO04402 | AGO04403 |
| *Ustilaginoidea virens* RNA virus 3 | UvV3 | YP_009004156 | YP_009004155 |
| *Verticillium dahliae* chrysovirus 1 | VdCV1 | ADG21213 | ADG21214 |
| *Verticillium dahliae* partitivirus 1 | VdPV1 | AGI52210 | AGI52209 |

**S1 Fig. Comparison of the amino acid sequences of the capsid protein (CP) of the *Pseudogymnoascus destructans* virus (PdPV-1), *Penicillium stoloniferum* virus S (PsV-S), *Gremmeniella abietina* virus MS1 (GaV-MS1), *Aspergillus ochraceus* virus (AoV), *Botryotinia fuckeliana* partitivirus-1 (BfPV1), *Aspergillus fumigatus* partitivirus-1 (AfuPV-1), *Ustilaginoidea virens* partitivirus 1 (UvPV-1), *Verticillium dahliae* partitivirus 1 (VdPV1), *Ophiostoma* partitivirus (OPV1), *Discula destructiva* virus 2 (DdV2), and *Fusarium solani* virus 1 (FusoV). Red: 100% identity; Blue: consensus match; Green: mismatch.**

**S2 Fig. Phylogenetic analyses of PdPV-1. Maximum likelihood phylogenetic tree based on CP amino acid sequences of representative members of the family *Partitiviridae, Totiviridae,* and *Chrysoviridae* were constructed using the program MEGA 6 (GenBank accession numbers are in the Table S2).**
